# Descriptive study of discharge medications in pediatric patients

**DOI:** 10.1177/2050312120927945

**Published:** 2020-06-03

**Authors:** Thao T Nguyen, Erica Bergeron, Teresa V Lewis, Jamie L Miller, Tracy M Hagemann, Stephen Neely, Peter N Johnson

**Affiliations:** 1 The Children’s Hospital at Saint Francis, Tulsa, OK, USA; 2 Department of Pharmacy: Clinical and Administrative Sciences, The University of Oklahoma College of Pharmacy, Oklahoma City, OK, USA; 3 University of Tennessee College of Pharmacy, Nashville, TN, USA

**Keywords:** Pediatrics, medication reconciliation, discharge, medication safety, dose rounding

## Abstract

**Background:**

Limited studies have evaluated medications in children discharged from hospitals. Knowledge of the number of medications and dosage forms could provide a baseline to establish a medication discharge prescription program.

**Objectives:**

To identify the median number of discharge prescriptions per patient. Secondary objectives included an evaluation of the dosage formulations and frequency, and comparisons of the prevalence of unrounded medication doses between service type (medical vs surgical) and physician provider level (trainees vs attendings).

**Methods:**

This retrospective study included children <18 years receiving >1 discharge prescription during 4 selected months over a 1-year time frame. Comparisons were made via Pearson’s chi-square tests, Fisher’s Exact tests, and Kruskal–Wallis nonparametric rank tests as appropriate with a priori *p* value of <0.05.

**Results:**

A total of 852 patients were evaluated, with most (78.8%) on a medical service. The median (interquartile range) number of new medications at discharge was 2 (1–3), with the median total number of discharge medications of 3 (2–6). There was no difference in the net change of the median number of home medications stopped and new medications started between service types. The majority (72.2%) received >1 oral liquid medications. There was no difference in prescribing rates per service type and provider level. There was a difference in the number of unrounded doses between trainees versus attendings, 17.8% versus 9.5%, *p* = 0.048.

**Conclusion:**

Patients were discharged on a median of three medications, and most received >1 oral liquid medications. These data can be used to target children who would benefit from medication discharge prescription programs.

## Background

Numerous studies have documented the risk of medication errors in the in-patient setting for pediatric patients.^1–6^ Several of these studies have documented that dosing and administration errors are the most common type of medication errors in children.^1–3^ Analgesics and antimicrobials are associated with the highest error rates.^3–6^ These errors have been attributed to several factors including calculation errors and lack of standardization between published dosing guidelines.^1,3,4^

Previous studies have primarily focused on errors that occur in the in-patient setting.^1–3,5^ However, few studies have evaluated the potential errors that occur during transitions of care among children discharged from the hospital. One recent study found that 80% of pediatric in-patient discharge prescriptions had >1 prescribing error.^7^ Providers at most children’s hospitals utilize computerized prescriber order entry (CPOE) systems to prescribe medications during the in-patient setting as well as at the time of hospital discharge. While CPOE has reduced the number of calculation errors for weight-based dosing in children, it may result in a different type of dosing error when unreasonable and unmeasurable doses are calculated (e.g. 50.143 vs 50 mg).^8^ If prescribed, unmeasurable doses, may also contribute to caregiver administration errors of oral liquid medications. For example, if a child is prescribed an amoxicillin suspension (400 mg/5 mL) with a dose of 333 mg, the resulting volume would be 4.16 mL. Caregivers may have difficulty in administering the correct dose given the standard markings on commonly utilized oral syringes.

Currently, limited studies have evaluated the most common medications and prevalence of unrounded doses prescribed to children upon hospital discharge.^7,9–11^ One recent study noted that children with multiple maintenance medications had an increased number of medication discrepancies during transitions of care.^12^ The purpose of this descriptive study was to quantify and describe discharge prescriptions in hospitalized children.

## Methods

This cross-sectional, retrospective, cohort study included children <18 years if they received >1 discharge prescription during January, April, July, or October, during a 1-year time frame (2014) at a 314-bed tertiary-care, academic children’s hospital. These 4 months were selected to serve as a representative month for each of the four seasons, to account for seasonal variation in disease state presentation and associated medication use. For example, in the United States, respiratory infections may be more predominant in the winter months, and asthma exacerbations may be common in the fall and spring. As a result, the types of discharge medications would vary from season to season based on the reason for admission. After Institutional Review Board approval, patients were identified within the CPOE system. If patients were discharged >1 time during the study period, each discharge encounter was counted separately. Patients without discharge medications were excluded.

Demographic data collected included age, home medications, and primary service provider. The providers were classified as attending physicians, physician trainees (i.e. fellows or residents), or non-physician provider. Discharge prescription data included the number of medications prescribed at discharge, medication frequency (i.e. maintenance vs as needed (PRN)), dosage formulations (e.g. liquids, capsules, tablets), and need for extemporaneous preparation for oral liquids. Each medication was categorized as 1 of 24 classes according to the American Hospital Formulary Services (AHFS) Pharmacologic Therapeutic Classification.^13^

The primary objective was to identify the median number of discharge prescriptions per patient. Secondary objectives included an evaluation of the dosage formulation, frequency, and AFHS system.^13^ Additional secondary objectives included a comparison of the top four AHFS classes and prevalence of unrounded medication doses between the service type and physician provider level. The service type was differentiated into surgical versus medical services to account for potential differences in prescribing practices, disease states, and patient populations between these two groups. For the provider level, only the trainee and attending physicians’ prescriptions were compared in order to delineate the impact of level of experience. Non-physician discharge prescriptions were excluded from this analysis as this allowed for a direct comparison of trainee status for prescribers in the same discipline. Doses were categorized as appropriately rounded or unrounded based on the definition consistent with Jones et al.^8^ and was defined as (1) unrounded dose calculated to <0.1 unit (e.g. mg, mcg) for non-neonatal intensive care unit (non-NICU) patients and a dose calculated to <0.01 unit (e.g. mg, mcg) for NICU patients and (2) an unrounded volume per dose defined as the corresponding volume of medication calculated to <0.1 mL for non-NICU patients and volume dose calculated to <0.01 mL for NICU patients.

## Statistical analyses

Data were collected and managed using Research Electronic Data Capture (REDCap) electronic data capture tools.^14^ Categorical variables, including demographics between admission months and differences between service type and physician provider level, were compared using asymptotic Pearson’s chi-square tests or Fisher’s Exact tests, as appropriate. As median (interquartile (IQR)) ranges were utilized to summarize continuous data, Kruskal–Wallis nonparametric rank tests were used to compare demographics between admission months and differences between service types. Post hoc tests were performed using Steel–Dwass–Critchlow–Fligner nonparametric method for all pairwise comparisons if the Kruskal–Wallis test was found significant. Analyses were conducted using SAS software v9.4 for Windows (SAS Institute.; Cary, North Carolina).

## Results

Demographics for the 852 patients with >1 discharge medication described in Table 1. October had the highest number of discharges (*n* = 389), accounting for 45% in this cohort. In the overall population, the majority (52.2%) were male at a median age of 4 years. Most patients (*n* = 671; 78.8%) were discharged from a medical service with a majority from General Pediatrics (*n* = 421; 62.7%), followed by the Hematology/Oncology (*n* = 125; 18.6%) and Gastroenterology teams (*n* = 30; 4.5%). There were 181 patients (21.2%) discharged from a surgical service, with the most common being Pediatric Surgery (*n* = 160; 88.4%) and Orthopedics (*n* = 6; 3.3%). Physicians prescribed 49.4% of discharge medications, including physician trainees (*n* = 369; 87.6%) and attending physicians (*n* = 52; 12.4%). The remaining 50.6% were prescribed by non-physician providers. The overall hospital length of stay (LOS) was a median (IQR) of 3 days (2–6). An overall difference in LOS was noted between the 4 months (*p* = 0.013) with January’s median stay (4.5 days) being longer than April (3.0 days, *p* = 0.049), July (3.0 days, *p* = 0.014) and October (3.0 days, *p* = 0.018). No other pairwise comparisons significantly differed.

**Table 1.** Baseline demographics (*n* = 852).

Characteristics	January (*n* = 74)	April (*n* = 146)	July (*n* = 243)	October (*n* = 389)	*p* value
Males, no. (%)	35 (47)	90 (62)	118 (49)	202 (52)	0.065^1^
Age (years), median (IQR)	2.00 (0.75–9)	3.00 (0.87–11)	5.00 (1–11)	5.00 (1–11)	0.444^2^
Hospital length of stay (days), median (IQR)	4.5 (3–9)	3 (2–7)^a^	3 (2–6)^a^	3 (2–6)^a^	0.013^2^

1 Asymptotic Pearson’s chi-square test.

2 Kruskal–Wallis one-way test.

a Differed significantly from January using Steel–Dwass–Critchlow–Fligner pairwise comparison procedure.

The overall median (IQR) number of home medications upon admission was 1 (1–4), whereas new medications at discharge was 2 (1–3) and total number of discharge medications of 3 (2–6). The median number of discharge medications between January, April, July, and October was 4 (2–7), 4 (2–6), 3 (1–5), and 3 (2–6). The only statistically significant difference between calendar months was April versus July (*p* = 0.031) and January versus July (*p* = 0.011). Patients on the medical services had a higher median number of home medications discontinued upon admission than those on surgical services, 2 (1–2) versus 1.5 (1–2), *p* < 0.01. In addition, patients on the medical service had a higher median number of new medications added at discharge than those on surgical services, 2 (1–3) versus 2 (1–2), *p* < 0.01. Despite this, there was no difference in the overall net change of home medications stopped and new medications added between the medical versus surgical services, 1 (0–2) versus 1 (1–2), *p* = 0.621.

There were 3427 total discharge prescriptions. Table 2 provides an overview of medication frequency, dosage formulations, and AHFS classes. The majority (82.6%) were discharged on a maintenance medication with a median (IQR) of 3 (1–4) per patient; most (59.9%) also received at least one PRN medication, with a median of 1 (1–3). When analyzing the medications based on dosage formulations, most children received either a liquid medication or capsule/tablet, with a limited number of children receiving suppositories or injectable medications (Table 2). The majority (*n* = 615; 72.2%) received either a commercially available or extemporaneously prepared liquid medication, with a median of 2 (1–3) and 1 (1–1) medication(s) per patient, respectively. The most common AHFS class was central nervous system (CNS) agents (51.1%), with patients receiving a median of 2 (1–2). The majority (*n* = 306; 70.3%) of these patients received >1 analgesic. The next three most common AHFS classes prescribed were anti-infectives (44.0%), gastrointestinal (43.7%), and hormones and synthetic substitutes (24.3%). There was variability between the prescribing of these AHFS classes between trainees versus attending physicians and medical versus surgical services (Table 3).

**Table 2.** Discharge prescriptions by frequency, dosage form, and American Hospital Formulary Service classifications.

Variable	No. (%) of patients receiving (*n* = 852)	Median (IQR) per patient receiving (row *n* noted below)
*Medication frequency*		
Maintenance	704 (82.6)	3 (1–4)
PRN	510 (59.9)	1 (1–3)
*Medication dosage formulations*		
Commercially available liquid medications	504 (59.2)	2 (1–3)
Capsules or tablets	421 (49.4)	2 (1–4)
Extemporaneously prepared liquid medications	111 (13.0)	1 (1–1)
Injectable medications	49 (5.8)	1 (1–2)
Suppositories	15 (1.8)	1 (1–1)
*AHFS classifications*		
Anti-histamine agents	122 (14.3)	1 (1–1)
Anti-infective agents	375 (44.0)	1 (1–2)
Anti-neoplastic agents	57 (6.7)	1 (1–1)
Autonomic agents	190 (22.3)	1 (1–1)
Blood derivative agents	1 (0.1)	1 (1–1)
Blood formation, coagulation, and thrombosis agents	72 (8.5)	1 (1–1)
Cardiovascular agents	109 (12.8)	1 (1–2)
Central nervous system agents	435 (51.1)	2 (1–2)
Contraceptive agents	1 (0.1)	1 (1–1)
Electrolytic, caloric, and water balance agents	101 (11.9)	1 (1–2)
Eye, ear, nose, and throat preparation agents	76 (8.9)	1 (1–1)
Gastrointestinal agents	372 (43.7)	1 (1–2)
Hormones and synthetic substitute agents	207 (24.3)	1 (1–2)
Local anesthetic agents	7 (0.8)	1 (1–1)
Respiratory tract agents	64 (7.5)	1 (1–1)
Skin and mucous membrane agents	87 (10.2)	1 (1–1)
Smooth muscle relaxant agents	7 (0.8)	1 (1–1)
Vitamins	99 (11.6)	1 (1–1)

PRN: as needed medication; AHFS: American Hospital Formulary Service.

**Table 3.** Prescribing trends by service and physician provider level for the top four common American Hospital Formulary Service classes at hospital discharge.

	Anti-infectives		CNS agents		Gastrointestinal drugs		Hormones and synthetic substitutes	
	No. (%)	Median (IQR)	No. (%)	Median (IQR)	No. (%)	Median (IQR)	No. (%)	Median (IQR)
Service type (*n*):								
Medical (*n* = 671)	334 (49.8)	1 (1–1)	285 (42.5)	2 (1–2)	312 (46.5)	1 (1–2)	189 (28.2)	1 (1–2)
Surgical (*n* = 181)	41 (22.7)	1 (1–2)	150 (82.9)	2 (1–2)	60 (33.1)	1 (1–2)	18 (9.9)	1 (1–1)
Physician provider level (*n*):								
Trainee (*n* = 369)	151 (40.9)	1 (1–1)	138 (37.4)	2 (1–2)	128 (34.7)	1 (1–2)	102 (27.6)	1 (1–2)
Attending (*n* = 52)	25 (48.1)	1 (1–1)	16 (30.8)	2 (1–2)	20 (38.5)	1 (1–1)	17 (32.7)	1 (1–2)
*Medical service (n)*								
Bone marrow transplant (*n* = 2)	2 (100)	2 (1–3)	2 (100)	1 (1–1)	2 (100)	2.5 (2–3)	–	–
Cardiology (*n* = 24)	9 (37.5)	1 (1–1)	15 (62.5)	1 (1–2)	10 (41.7)	1 (1–1)	1 (4.2)	1 (1–1)
Endocrinology (*n* = 13)	–	–	1 (7.7)	1 (1–1)	–	–	13 (100)	2 (2–2)
Family medicine (*n* = 2)	–	–	1 (50)	1 (1–1)	1 (50)	1 (1–1)	–	–
Gastroenterology (*n* = 30)	10 (33.3)	1 (1–2)	8 (26.7)	2 (1.5–2.5)	22 (73.3)	1 (1–2)	8 (26.7)	1 (1–1)
General pediatrics (*n* = 421)	176 (41.8)	1 (1–1)	154 (36.6)	2 (1–2)	148 (35.2)	1 (1–2)	119 (28.3)	1 (1–2)
Hematology and oncology (*n* = 125)	116 (92.8)	1 (1–1)	94 (75.2)	2 (1–3)	107 (85.6)	2 (2–3)	25 (20)	1 (1–1)
Nephrology (*n* = 25)	11 (44)	2 (1–3)	5 (20)	1 (1–2)	10 (40)	1.5 (1–2)	14 (56)	1 (1–1)
NICU (*n* = 18)	6 (33.3)	1 (1–1)	3 (16.7)	1 (1–1)	6 (33.3)	1 (1–2)	–	–
Pulmonology (*n* = 11)	4 (36.4)	1.5 (1–2.5)	2 (18.2)	3 (1–5)	6 (54.5)	1 (1–2)	9 (81.8)	2 (2–2)
*Surgical service (n)*								
Pediatric surgery (*n* = 160)	33 (20.6)	1 (1–2)	133 (83.1)	1 (1–2)	52 (32.5)	1 (1–2)	13 (8.1)	1 (1–1)
Neurosurgery (*n* = 1)	–	–	1 (100)	2 (2–2)	–	–	–	–
Orthopedics (*n* = 6)	2 (33.3)	1 (1–1)	5 (83.3)	2 (1–4)	2 (33.3)	1 (1–1)	–	–
Otorhinolaryngology (*n* = 4)	1 (25)	1 (1–1)	3 (75)	2 (2–3)	3 (75)	2 (2–3)	2 (50)	1 (1–1)
Solid organ transplant (*n* = 2)	2 (100)	3.5 (3–4)	2 (100)	1.5 (1–2)	2 (100)	2 (2–2)	2 (100)	1 (1–1)
Urology (*n* = 7)	3 (42.9)	1 (1–3)	6 (85.7)	2 (2–2)	1 (14.3)	3 (3–3)	1 (14.3)	1 (1–1)

CNS: Central nervous system agents; NICU: neonatal intensive care unit.

There were 1233 liquid medications prescribed to 615 patients, and 154 (12.5%) of these medications were unrounded. There was no significant difference between medical versus surgical services that prescribed an unmeasurable dose of a liquid medication at discharge, 12.2% versus 14.3%, *p* = 0.417. However, there was a significant difference in the number of trainees versus attending physicians, 17.8% versus 9.5%, *p* = 0.048.

## Discussion

This is the first study to evaluate the number and type of medications that hospitalized children received at discharge. Previous studies have evaluated discharge prescription review programs, but few have qualified the type of medications that children received.^7,9–11^ The majority (79%) from our study were discharged from a medical service, and 82.6% were discharged on a maintenance medication. The median (IQR) number of total discharge medications was 3 (2–6), but there was no difference in the overall net change of home medications stopped and new medications added at discharge. The purpose of this descriptive study was to utilize this information to help our institution develop a program that would aid in transitions of care. Currently, our institution does not have a formal medication discharge prescription program (MDPP); other institutions have implemented a program and found positive effects on medication errors and cost-savings.^7,10,11^

Most patients were discharged from a medical service, with the primary service being General Pediatrics (62.7%). During the study, there were four General Pediatrics teams, three were teaching teams and one staffed by pediatric attendings. This finding was consistent with a study by Huynh et al.^9^ who conducted a prospective, multi-center study over a 5-month period in 244 children who received >1 medication and noted that General Pediatrics had the most discharges.^9^ We also noted that 50.6% of discharge medications were prescribed by non-physician providers, such as physician associates/nurse practitioners. At our institution, these non-physician providers care for children on specialty medical services like Gastroenterology, Cardiology, and Hematology/Oncology. The remaining medications were prescribed by physicians, with the majority being physician trainees (87.6%). A comparison was made between attendings and trainee physicians to identify if level of experience impacted the selection of unrounded or appropriate dose of an oral liquid medication. However, we did not compare differences in discharge medications between non-physician and physician providers as it would be difficult to quantify the level of training and experience for non-physician providers who may have had previous work experience to ensure an adequate comparator group. We did not analyze the frequency or types of medication errors, but these data provide a baseline since previous studies have noted increased medication errors with physician trainees versus attendings.^15,16^

The median (IQR) number of home medications for the overall patient population was 1 (1–4) with the median number of new medications at discharge of 2 (1–3), for a median of 3 total medications (2–6) at discharge. In addition, patients on medical services had a higher number of medications added at discharge compared with those on surgical services, 2 (1–3) versus 2 (1–2), *p* < 0.01. This finding is consistent with Christiansen et al.^7^ who evaluated a pharmacist-led prescription review program over a 30-day period and found that the mean number of discharge prescriptions was 3, range 1–9. Similar to Christiansen et al.,^7^ we did not stratify these medications based on the medical complexity of the children. As the number of medically complex children in the hospital setting increase, this could result in a greater average number of discharge medications. Approximately 19.8% of children <18 years have special health care needs, requiring a mean number of 5–9 medications.^12,17,18^ It is probable that these children may be initiated on additional medications at discharge. As a result, some studies have noted that medically-complex children would be at increased risk of medication errors.^18^

As noted, 3427 medications were prescribed at discharge, with 82.6% of children receiving >1 maintenance medication. The two most common AHFS classes were CNS agents (51.1%) and anti-infectives (44.0%). Several studies have evaluated the top medications classes prescribed for children and found similar results with the most common being analgesics/sedatives and anti-infectives, which are also noted to have increased medication errors.^3,5,9^

To our knowledge, no prior study has evaluated discharge medication formulations in children. We noted that children received a number of different dosage formulations including commercially available or extemporaneously prepared liquid medications, capsules/tablets, injectable medications, and suppositories. Most children received either a liquid medication or capsule or tablet. As this is a single-center study, it is difficult for us to compare our findings on the variation of dosage forms compared to other health-systems. The majority (72.2%) received >1 commercially available or extemporaneously prepared liquid medication. Yin et al.^19^ evaluated the association between the type of dosing unit used for liquid medications (i.e. mL vs teaspoon/tablespoon) and resulting medication errors by caregivers in 287 children discharged from an emergency department; they found that 41.1% of caregivers made an error and had two-times higher odds for errors when prescribed as teaspoon/tablespoon versus mLs (45.1% vs 31.4%, *p* = 0.04; adjusted odds ratio = 1.9; 95% Confidence Interval (CI):1.03–3.5). We found that 12.5% of liquid medications were unrounded and not easily measurable using a standard oral syringe. Difficulties in accurate measurement and potential risk for further error could occur if unrounded doses are prescribed.

Currently, our institution does not standardize pharmacist responsibility at the time of discharge. The American Academy of Pediatrics recommends hospitals utilize clinical pharmacists with postgraduate training in pediatric pharmacy on multidisciplinary healthcare teams, as they play an integral role in the medication reconciliation process.^20^ Previous studies have described the role of pediatric pharmacists on outcomes of discharged patients.^7,10,11^ Nguyen et al.^11^ evaluated the impact of an interprofessional medication discharge program involving a nurse and pharmacist over a 5-month period; the three most common interventions involved clarification of medication orders, assistance in obtaining medications, and dose rounding. As noted, we found that 12.5% of all liquid medications were unrounded and there was a greater number of unrounded liquid medications between trainees versus attending physicians, 17.8% versus 9.5%, *p* = 0.048. It is possible that implementation of a pediatric pharmacist’s review of discharge medications would have resulted in identification of these potential errors prior to discharge. In addition, creation of a CPOE rule could force providers to select a rounded dose. Medication counseling at the time of discharge was performed in some studies assessing the impact of pediatric pharmacists.^7,10,11^ In 2014, the National Council for Prescription Drugs Programs issued a White Paper regarding the best practice of dispensing oral liquid medications for community pharmacists.^21^ They recommended that best practices include counseling for caregivers on appropriate administration and instruction on proper dosing devices. It is feasible that involving the pharmacist prior to discharge may identify medication doses that are easily measurable. Pharmacists can work with other members of the healthcare team to provide more in-depth patient counseling sessions that can focus on reviewing adherence concerns with home medications. This may be especially helpful for patients who may need reinforcement of administration techniques for medications like albuterol and insulin to prevent patients from being readmitted from conditions like asthma and diabetes. They could also utilize the teach-back method to help address understanding of new medications and ensure the caregiver’s ability to accurate measure and administer medication doses.^22^

Despite the positive benefits of pharmacists in the medication discharge process, it may be difficult to implement such a service for all patients. As every institution has varying levels of acuity, we would recommend to conduct a descriptive project such as the present study to establish a baseline and identify potential opportunities for improvement between service types and providers. In addition, institutions could utilize the literature for guidance to identify high-risk patients for medication errors at discharge. DeCourcey et al.^12^ performed a prospective observational study in 308 patients <25 years with a chronic disease to determine factors associated with medication discrepancies. They noted in their multivariable analyses that each additional home medication (adjusted rate ratio (ARR) 1.07 (95% CI:1.04–1.10)) and chronic respiratory medications (ARR 1.51 (95% CI:1.01–2.28)) were associated with increased discrepancies. Therefore, a prudent approach may be to target children with >5 maintenance medications, as previous studies indicated that medically complex children require 5–9 medications.^12,18^ Pharmacists could also be consulted in children prescribed >1 extemporaneously prepared liquid medication. As noted, 13% received a liquid medication that was not commercially available. This is important since there can be significant variability in extemporaneous formulas compounded by in-patient versus out-patient pharmacies.^23^

There are several limitations with this study. First, this was a retrospective study that focused on 4 months during a 1-year time frame. There were 852 patients discharged with >1 medication out of approximately 13,000 admissions during the 1-year time frame, accounting for 6.6% of admissions. To address this, we included patients discharged during each quarter to account for seasonal changes. Second, there is no standard definition of unrounded medication doses. We utilized a definition from our previous work for comparison.^8^ Third, we did not assess each discharge prescription for the likelihood of a medication error. With the retrospective design, it is impossible to accurately check each medication dose without clear understanding of the indication for each patient. We utilized this descriptive study to identify baseline information on the number of medications, AHFS classes, formulations, unrounded doses of liquid medications, and comparisons of these data between service and physician provider level. Future studies should focus on the impact of a standardized pharmacist involvement on the impact of an MDPP.

## Conclusion

In this study, children received a median of three discharge medications, but there was no difference in the overall net change of home medications stopped and new medications added between medical versus surgical services. Most received >1 oral liquid medication, with 13% requiring an extemporaneous preparation. The top four AHFS classes for discharge prescriptions included CNS, anti-infectives, gastrointestinal agents, and hormones and synthetic substitutes. These data can be used to target children who would benefit from an MDPP.

## References

[1] Wong IC Ghaleb MA Franklin BD, et al Incidence and nature of dosing errors in paediatric medication: a systematic review. Drug Safety 2004; 27(9): 661–670. 15230647 10.2165/00002018-200427090-00004

[2] Stucky ER, American Academy of Pediatrics Committee on Drugs; American Academy of Pediatrics Committee on Hospital Care. Prevention of medication errors in the pediatric inpatient setting. Pediatrics 2003; 112(2): 431–436. 12897304 10.1542/peds.112.2.431

[3] Kaushal R Bates DW Landrigan C, et al Medication errors and adverse drug events in pediatric inpatients. JAMA 2001; 285(16): 2114–2120. 11311101 10.1001/jama.285.16.2114

[4] McPhillips HA Stille CJ Smith D, et al Potential medication dosing errors in outpatient pediatrics. J Pediatr 2005; 147(6): 761–767. 16356427 10.1016/j.jpeds.2005.07.043

[5] Ghaleb MA Barber N Franklin BD, et al Systematic review of medication errors in pediatric patients. Ann Pharmacother 2006; 40(10): 1766–1776. 16985096 10.1345/aph.1G717

[6] Ross LM Wallace J Paton JY. Medication errors in a pediatric teaching hospital in the UK: five years operational experience. Arch Dis Child 2000; 83(6): 492–497. 11087283 10.1136/adc.83.6.492 PMC1718567

[7] Christiansen SR Morgan JA Hilmas E, et al Impact of a prescription review program on the accuracy and safety of discharge prescriptions in a pediatric hospital setting. J Pediatr Pharmacol Ther 2008; 13(4): 226–232. 23055881 10.5863/1551-6776-13.4.226 PMC3461987

[8] Jones AN Miller JL Neely S, et al Prevalence of unrounded medication doses and associated factors among hospitalized pediatric patients. J Pediatr Pharmacol Ther 2017; 22(4): 286–292. 28943824 10.5863/1551-6776-22.4.286 PMC5562209

[9] Huynh C Wong IC Tomlin S, et al An evaluation of paediatric medicines reconciliation at hospital discharge into the community. Int J Pharm Pract 2016; 24(3): 196–202. 26670624 10.1111/ijpp.12229

[10] Leathers LA Brittain KL Crowley K Effect of a pediatric prescription medication discharge program on reducing hospital readmission rates. J Pediatr Pharmacol Ther 2017(2); 22: 94–101. 28469533 10.5863/1551-6776-22.2.94 PMC5410864

[11] Nguyen V Sarik DA Dejos M, et al Development of an interprofessional pharmacist-nurse navigation pediatric discharge program. J Pediatr Pharmacol Ther 2018(4); 23: 320–328. 30181724 10.5863/1551-6776-23.4.320 PMC6117806

[12] DeCourcey DD Silverman M Chang E, et al Medication reconciliation failures in children and young adults with chronic disease during intensive and intermediate care. Pediatr Crit Care Med 2017; 18(4): 370–377. 28198758 10.1097/PCC.0000000000001090 PMC5380513

[13] McEvoy GK Snow ED Miller J, et al (eds) AHFS: drug information. 2019 ed Bethesda, MD: American Society of Health-system Pharmacists, 2019.

[14] Harris PA Taylor R Thielke R, et al Research electronic data capture (REDCap): a metadata-driven methodology and workflowprocess for providing translational research informatics support. J Biomed Inform 2009; 42(2): 377–381. 18929686 10.1016/j.jbi.2008.08.010 PMC2700030

[15] Condren MC Honey BL Carter SM, et al Influence of a systems-based approach to prescribing errors in a pediatric resident clinic. Acad Pediatr 2014; 14(5): 485–490. 25169160 10.1016/j.acap.2014.03.018

[16] Condren M Studebakerm IJ John BM. Prescribing errors in a pediatric clinic. Clin Pediatr 2010; 49(1): 49–53. 10.1177/0009922809342459 19643978

[17] Child Adolescent Health Measurement Initiative (2012). Who are children with special health care needs (CSHCN). Data resource center, supported by cooperative agreement 1-U59-MC06980-01 from the U.S. Department of Health and Human Services, Health Resources and Services Administration (HRSA), Maternal and Child Health Bureau (MCHB), http://www.childhealthdata.org (accessed 8 April 2020).

[18] Stone BL Boehme S Mundorff MB, et al Hospital admission reconciliation in medically complex children: an observational study. Arch Dis Child 2010; 95(4): 250–255. 19948664 10.1136/adc.2009.167528

[19] Yin HS Dreyer BP Ugboaja DC, et al Unit of measurement used and parent medication dosing errors. Pediatrics 2014(2); 134: 354–361. 10.1542/peds.2014-0395 PMC4187234 25022742

[20] Mueller BU Neuspiel DR Stucky Fisher ER, et al Principles of pediatric patient-safety: reducing harm due to medical care. Pediatrics 2019; 143(2): e20183649. 30670581 10.1542/peds.2018-3649

[21] National Coordinating Council for Medication Error Reporting Prevention. Index for categorizing medication errors, http://www.nccmerp.org/sites/default/files/indexColor2001-06-12.pdf (accessed 8 April 2020).

[22] Bertakis KD. The communication of information from physician to patient: a method for increasing patient retention and satisfaction. J Fam Pract 1977; 5(2): 217–222. 894226

[23] Engels MJ Ciarkowski SL Rood J, et al Standardization of compounded oral liquids for pediatric patients in Michigan. Am J Health Syst Pharm 2016; 73(13): 981–990. 27325879 10.2146/150471

